# Response: Commentary: Evolutionary conservation of acylplastoquinone species from cyanobacteria to eukaryotic photosynthetic organisms of green and red lineages

**DOI:** 10.3389/fpls.2025.1671717

**Published:** 2025-09-18

**Authors:** Ryo Ito, Mizuki Endo, Motohide Aoki, Shoko Fujiwara, Norihiro Sato

**Affiliations:** School of Life Sciences, Tokyo University of Pharmacy and Life Sciences, Hachioji, Japan

**Keywords:** targeted LC-MS/MS analysis, LC-MS/IDA-MS² analysis, acylplastoquinol, plastoquinone-B, cyanobacteria, red and green algae, haptophyte, seed plants

## 1 Introduction

Plastoquinone is the electron carrier in photosynthetic organisms, playing a crucial role in the photosynthetic electron transport chain. We previously reported the presence of two acylated plastoquinone species—acylplastoquinol (APQ) and plastoquinone-B (PQB)—in the unicellular cyanobacteria *Synechocystis* sp. PCC 6803 and *Synechococcus* sp. PCC 7002 (Kondo et al., 2023a, b). The gene *slr2103* and its ortholog *SYNPCC7002_A0918* encode a bifunctional acyltransferase responsible for the synthesis of both APQ and PQB in *Synechocystis* and *Synechococcus*, respectively (Kondo et al., 2023a, b). Orthologs of *slr2103* are found in over 100 cyanobacterial genomes but are absent in eukaryotic photosynthetic organisms (Kondo et al., 2023b). Building on these studies, the article reported that both APQ and PQB are present in two filamentous cyanobacteria containing *slr2103* orthologs, and also in a red alga, two green algae, a haptophyte, and a seed plant.

The commentary (Sato, 2025) on the article (Ito et al., 2025) raised issues regarding the identification methods of acylplastoquinone species and the research history of these compounds. Since the research history, including the initial identification of APQ in *Synechocystis* (Mori-Moriyama et al., 2023), is detailed in the commentary, this response will focus on the technical aspects.

## 2 Results and discussion

In the article, acylplastoquinone species were identified from total cellular lipids using profiling LC–MS analysis in ion-trap mode with information-dependent acquisition (IDA) MS² to analyze a broad range of compounds (retention time 2 to 18 min, m/z 300 to 1200; Ito et al., 2025). Identification was based on the m/z values of NH_4_^+^-adducted precursor ions, their retention times in the LC-MS chromatogram, and unique fragment ions in the MS² spectra (**Supplementary Table S1**; Kondo et al., 2023a, b). The relative contents of respective molecular species in APQ or PQB were estimated by measuring signal intensities of their corresponding lipid ions relative to total cellular lipid ions, revealing that APQ and PQB predominantly consist of saturated-acyl species. Importantly, this analytical approach allows for the detection of any substances that might interfere with the ionization of acylplastoquinone species (see below).

However, as noted in the commentary, fragment ions in the MS² spectra of acylplastoquinone species used for identification showed lower signal-to-noise (S/N) ratios (Ito et al., 2025) compared to those reported by Kondo et al. (2023b). The commentary suggested that low S/N ratios may result from low lipid content in the samples. Comparable total cellular lipid contents (based on cell mass, OD_730_·mL) routinely underwent LC-MS/IDA-MS² analysis in our studies, where similarly low S/N ratios were observed throughout the work by Kondo et al. (2023a, b). The difference likely arises from the MS analytical methods: unlike the profiling analysis by Ito et al. (2025), Kondo et al. (2023a, b) also performed targeted MS² analysis in ion-trap mode focusing on selected candidate ions of acylplastoquinone species, yielding improved S/N ratios.

**Figure 1:**
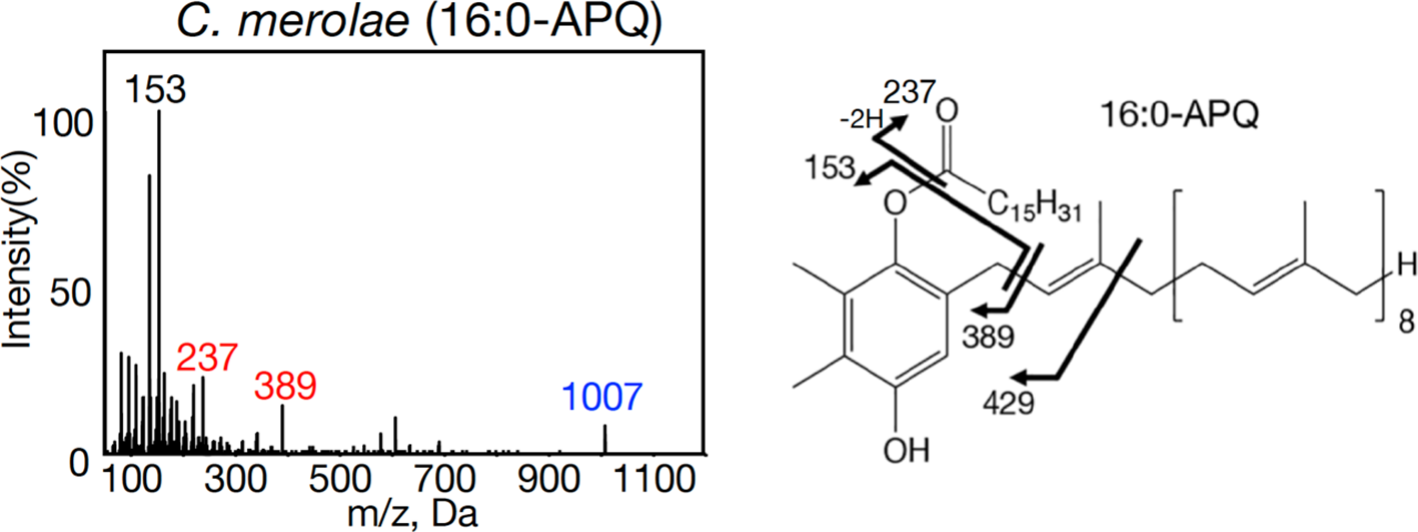
Representative MS^2^ spectra of 16:0-APQ with NH_4_^+^ adducts and its fragmentation patterns in the red alga *Cyanidioschyzon merolae*. Characteristic ions detected include m/z 153, m/z 237 (acyl-derived fragment), m/z 389 (de-prenylated fragment), and m/z 1007 (precursor).

Here, to evaluate our previous identification of acylplastoquinone species by profiling LC-MS/IDA-MS² analysis, we subjected samples identical to those used by Ito et al. (2025) to an alternative MS analysis. Using **Supplementary Table S1**, candidate acylplastoquinone species were searched via targeted LC-MS/MS analysis of total cellular lipids in multiple reaction monitoring (MRM) mode for enhanced selectivity and improved S/N ratios. Precursor ions of acylplastoquinone species were monitored under collision-induced dissociation at 50 eV, with transitions to m/z 153 for APQ and m/z 151 for PQB. Consequently, candidates of the same molecular species of APQ and PQB reported by Ito et al. (2025) were detected in their respective photosynthetic organisms. These candidates then underwent targeted MS² analysis in ion-trap mode with further optimization: collision-energy spread fine-tuning to confirm precursor and fragment ions, and dynamic fill time optimization to enhance overall ion signal intensity. The resulting MS² spectra showed diagnostic ions characteristic of acylplastoquinone species with high S/N ratios, consistent with those reported by Kondo et al. (2023a, b), strongly supporting our previous identification (**Figure 1**, **Supplementary Figures S1**-**S8**). Notably, MS² spectra for APQ were revised to include both de-prenylated and acyl-derived fragment ions (**Figure 1**, **Supplementary Figures S1**-**S3**), alongside a proposed model for generation of acyl-derived fragments within the chemical structure (**Figure 1**, **Supplementary Figure S7**), in response to the commentary. Moreover, both APQ and PQB included the precursor ions (**Figure 1**, **Supplementary Figures S1**-**S6**).

Regarding *Synechocystis*, Tanikawa et al. (2025), unlike Ito et al. (2025), detected 18:1-APQ. The commentary suggested potential interference by other lipids in the Ito et al. study; however, we did not detect any lipid ions that could potentially interfere within the m/z range of 300 to 1200 at retention times matching or close to that of 18:1-APQ in the profiling LC-MS chromatogram. The presence of 18:1-APQ in *Synechocystis* may depend on culturing conditions, including light intensity, temperature, aeration, culture vessel type, or growth stage. Meanwhile, according to the commentary, APQ content decreases during lipid concentration steps, including evaporation of lipid extracts, likely due to its susceptibility to oxidation. We are currently preparing new total lipid extracts from cells with or without an antioxidant in lipid extraction, to investigate the effects of its addition on APQ molecular species content. Establishing a reliable method for quantitative APQ analysis, as discussed as essential in the commentary, will require gradual accumulation of such data.

In conclusion, this study reinforces the validity of our earlier findings (Ito et al., 2025): APQ and PQB have been evolutionarily conserved from cyanobacteria to eukaryotic photosynthetic organisms in the green and red lineages. Moreover, under the culturing conditions used by Ito et al. (2025), these organisms predominantly contain saturated APQ and PQB species.
