# Supplementary material for: Response: Commentary: Evolutionary conservation of acylplastoquinone species from cyanobacteria to eukaryotic photosynthetic organisms of green and red lineages
Source: Front Plant Sci. 2025 Sep 18;16:1671717. doi: 10.3389/fpls.2025.1671717 (PMC12489937; doi:10.3389/fpls.2025.1671717)
Supplement: Supplementary file 1 [file DataSheet1.pdf]

Supplementary Table S1 LC–MS/MS detection parameters for APQ and PQB molecular species

Lipids	Precursors m/z (NH_4^+-adduct)	Diagnostic fragments m/z	Retention times (min)
14:0-APQ	m/z 979	m/z 153	*14.5*
16:0-APQ[a]	m/z 1007	m/z 153	15.2
17:0-APQ	m/z 1021	m/z 153	*15.6*
18:0-APQ[a]	m/z 1035	m/z 153	15.9
18:1-APQ	m/z 1033	m/z 153	*15.1*
14:0-PQB	m/z 993	m/z 151, 748	*14.5*
16:0-PQB[a]	m/z 1021	m/z 151, 748	15.6
18:0-PQB[a]	m/z 1049	m/z 151, 748	16.4
18:2-PQB	m/z 1045	m/z 151, 748	*15.1*

[a]For detection of 16:0- and 18:0-APQ ions and 16:0- and 18:0-PQB ones, previously reported parameter values were used as follows: the m/z values of NH_4^+-adducted lipid ions and the retention times of lipids in LC-MS chromatograms, and the m/z values of diagnostic fragment ions in MS^2 spectra (Kondo et al., 2023b). For the other APQ and PQB molecular species, the calculated m/z values of NH_4^+-adducted lipid ions, the postulated diagnostic fragment ions, and the retention times (shown in italics) determined in this study are presented.

.
