# Supplementary material for: Response: Commentary: Evolutionary conservation of acylplastoquinone species from cyanobacteria to eukaryotic photosynthetic organisms of green and red lineages
Source: Front Plant Sci. 2025 Sep 18;16:1671717. doi: 10.3389/fpls.2025.1671717 (PMC12489937; doi:10.3389/fpls.2025.1671717)
Supplement: Supplementary file 2 [file DataSheet2.pdf]

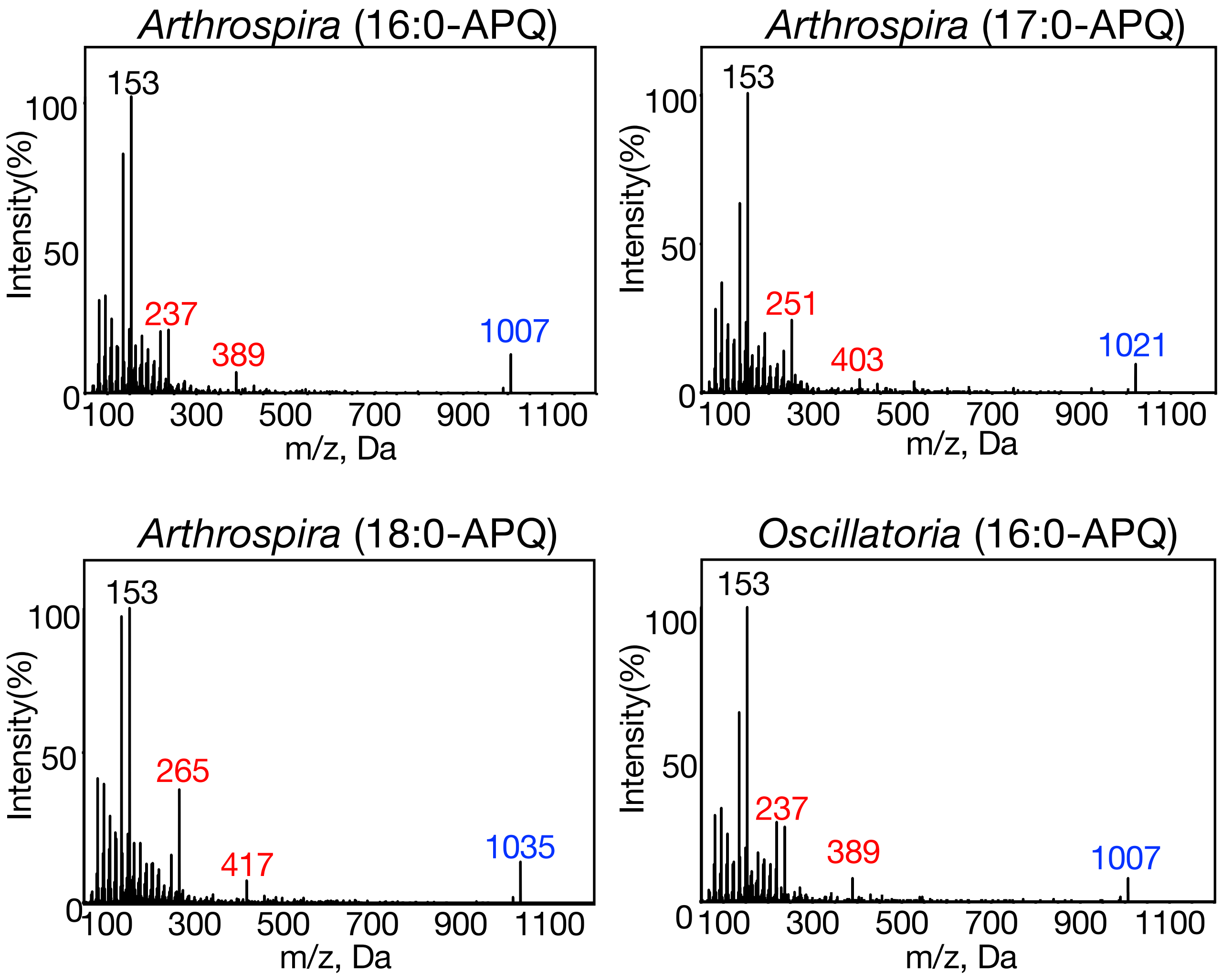

Figure S1 MS^2 spectra of APQ species with NH_4^+ adducts in cyanobacteria. *Arthrospira platensis*, 16:0-, 17:0-, and 18:0-APQ; *Oscillatoria rosea* Utermöhl, 16:0-APQ. Characteristic ions detected include m/z 153, acyl-derived and de-prenylated fragment ions (shown in red), and the precursor ion (shown in blue), as in Figure 1. Refer to Figure 7S for fragmentation patterns.

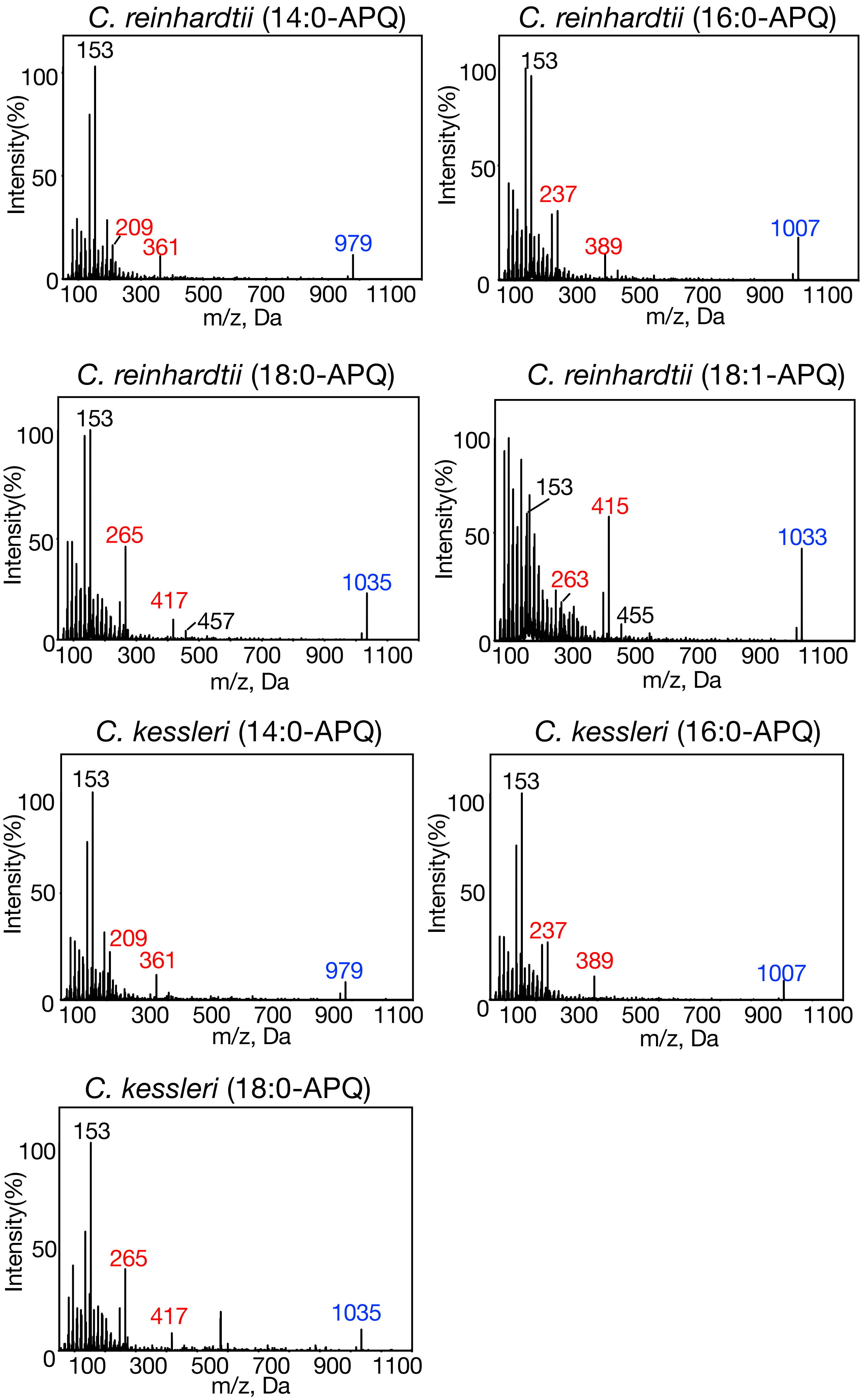
C. reinhardtii (14:0-APQ)
153
209
361
979
Intensity(%)
m/z, Da
C. reinhardtii (16:0-APQ)
153
237
389
1007
Intensity(%)
m/z, Da
C. reinhardtii (18:0-APQ)
153
265
417
457
1035
Intensity(%)
m/z, Da
C. reinhardtii (18:1-APQ)
153
415
263
455
1033
Intensity(%)
m/z, Da
C. kessleri (14:0-APQ)
153
209
361
979
Intensity(%)
m/z, Da
C. kessleri (16:0-APQ)
153
237
389
1007
Intensity(%)
m/z, Da
C. kessleri (18:0-APQ)
153
265
417
1035
Intensity(%)
m/z, Da

Figure S2 MS^2 spectra of APQ species with NH_4^+ adducts in green algae. *Chlamydomonas reinhardtii*, 14:0-, 16:0-, 18:0-, and 18:1-APQ; *Chlorella kessleri*, 14:0-, 16:0-, and 18:0-APQ. Characteristic ions detected include m/z 153, acyl-derived and de-prenylated fragment ions (shown in red), and the precursor ion (shown in blue), as in Figure 1. Refer to Figure 7S for fragmentation patterns.

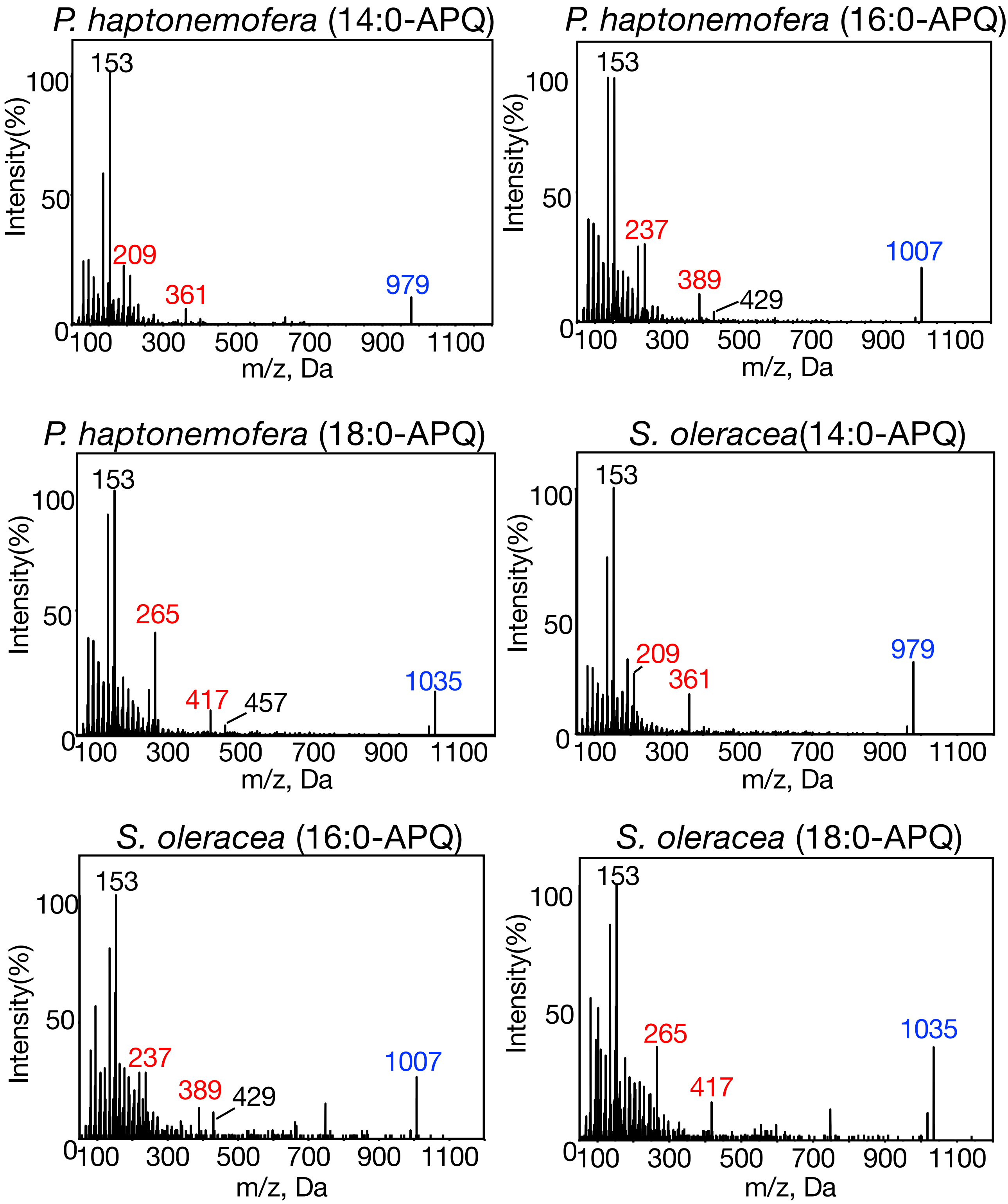

Figure S3 MS[2] spectra of APQ species with NH_4^+ adducts in a haptophyte, *Pleurochrysis haptonemofera* and a seed plant, *Spinacia oleracea*. *P. haptonemofera*, 14:0-, 16:0-, and 18:0-APQ; *S. oleracea*, 14:0-, 16:0-, and 18:0-APQ. Characteristic ions detected include m/z 153, acyl-derived and de-prenylated fragment ions (shown in red), and the precursor ion (shown in blue), as in Figure 1. Refer to Figure 7S for fragmentation patterns.

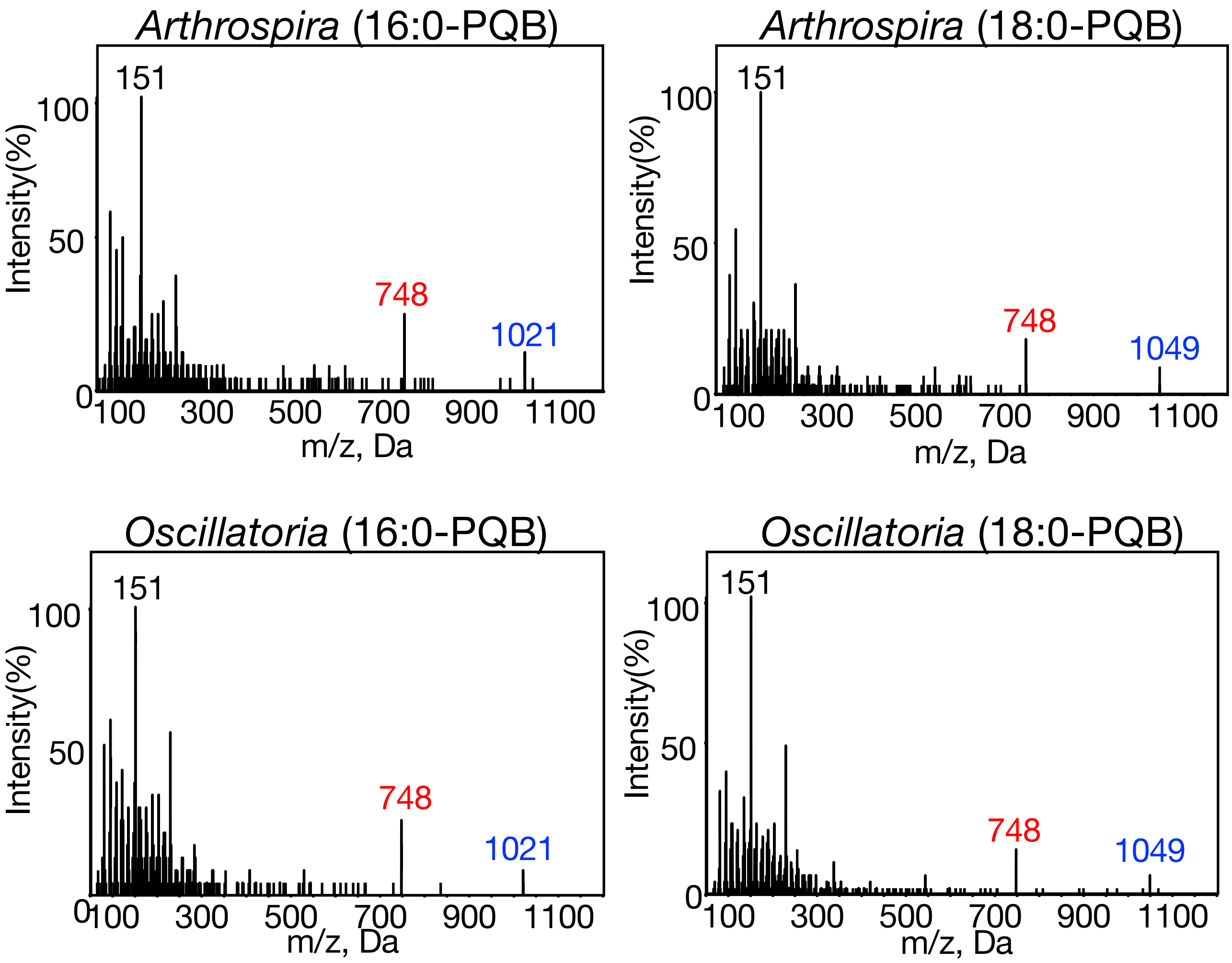

Figure S4 MS[2] spectra of PQB species with NH_4^+ adducts in cyanobacteria. *Arthrospira platensis*, 16:0- and 18:0-PQB; *Oscillatoria rosea* Utermöhl, 16:0- and 18:0-PQB. Characteristic ions detected include m/z 151, m/z 748 (deacylated fragment ion, shown in red), and precursor ion (shown in blue). Refer to Figure 8S for fragmentation patterns.

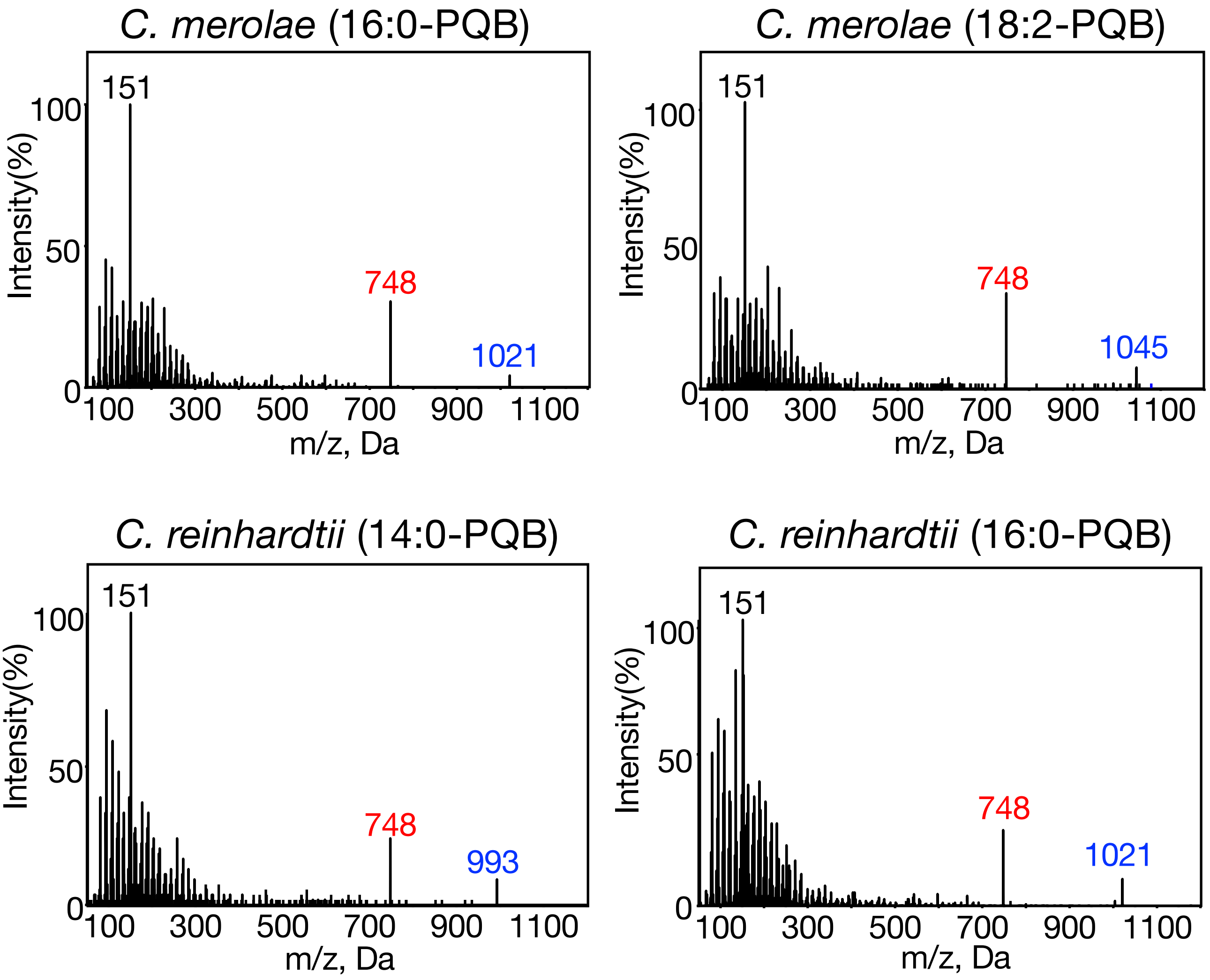

Figure S5 MS^2 spectra of PQB species with NH_4^+ adducts in red and green algae. *C. merolae*, 16:0- and 18:2-PQB; *C. reinhardtii*, 14:0- and 16:0-PQB. Characteristic ions detected include m/z 151, and m/z 748 (de-acylated fragment ion, shown in red), and precursor ion (shown in blue). Refer to Figure 8S for fragmentation patterns.

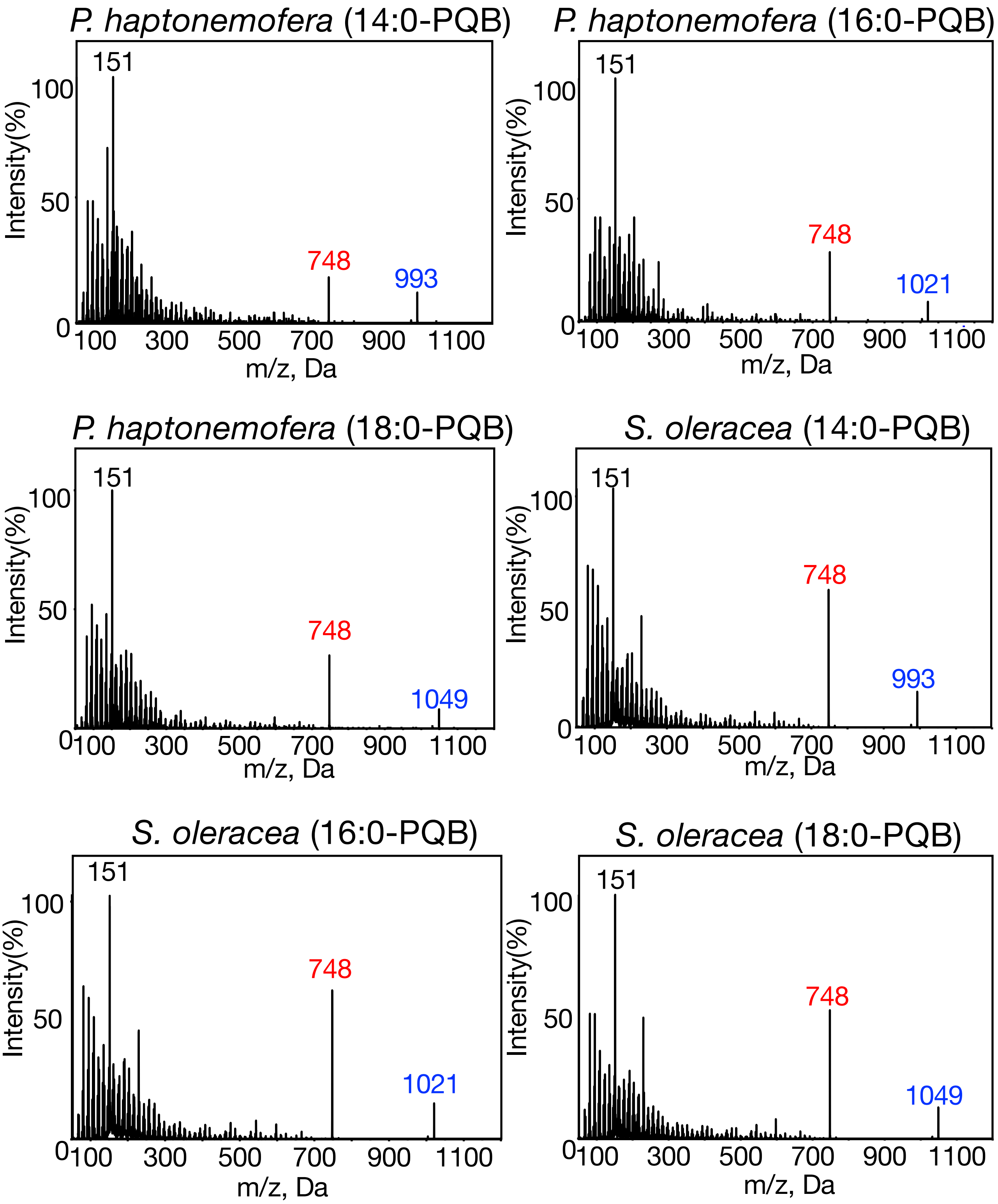

Figure S6 MS² spectra of PQB species with NH_4^+ adducts in a haptophyte, *Pleurochrysis haptonemofera* and a seep plant, *Spinacia oleracea*. *P. haptonemofera*, 14:0-, 16:0-, and 18:0-PQB; *S. oleracea*, 14:0-, 16:0-, and 18:0-PQB. Characteristic ions detected include m/z 151, and m/z 748 (de-acylated fragment ion, shown in red), and precursor ion (shown in blue). Refer to Figure 8S for fragmentation patterns.

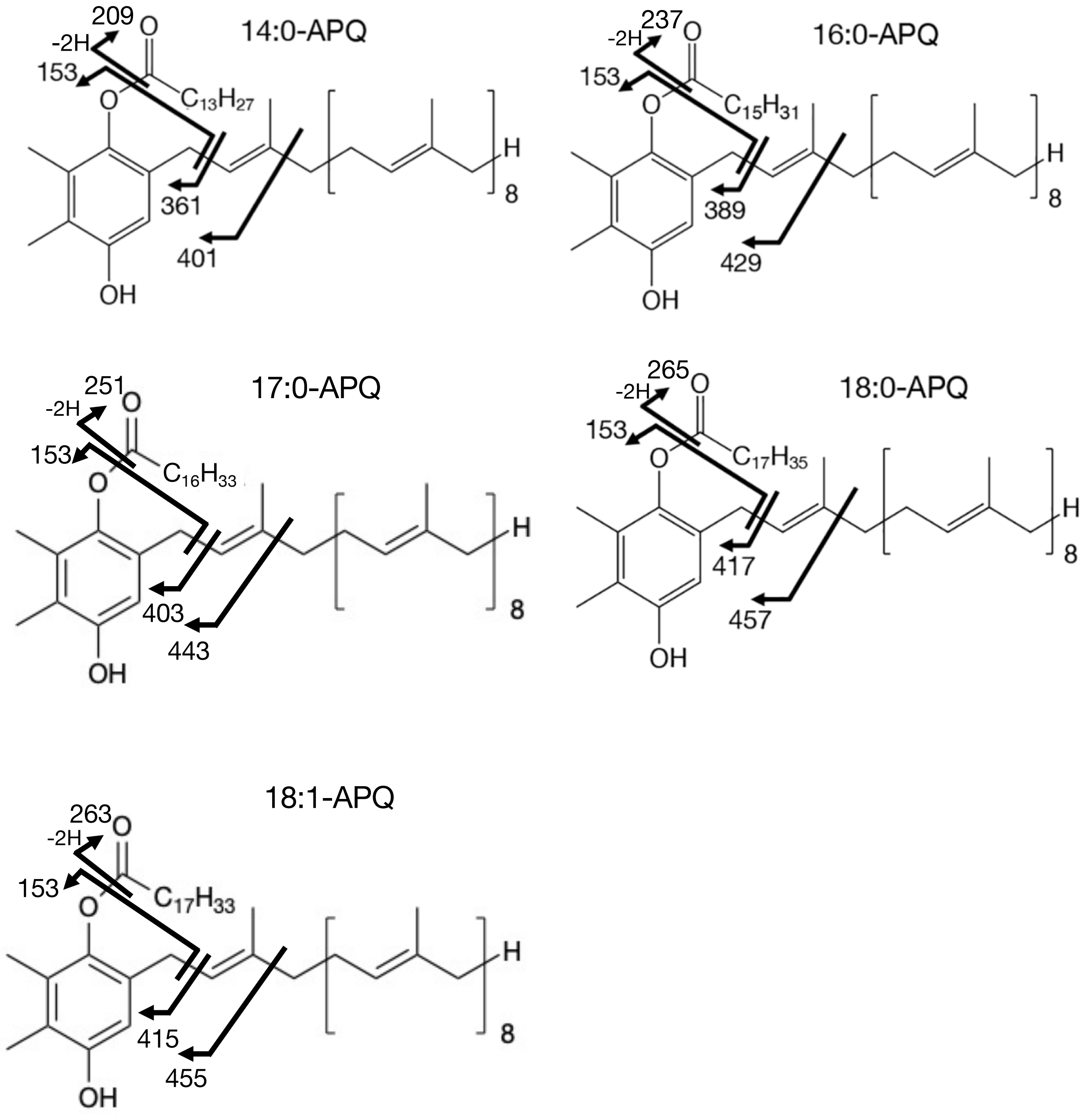

Figure S7 Fragmentation patterns of APQ species. Note that 2H are lost for generation of acyl-derived fragment ions in respective species.

14:0-PQB

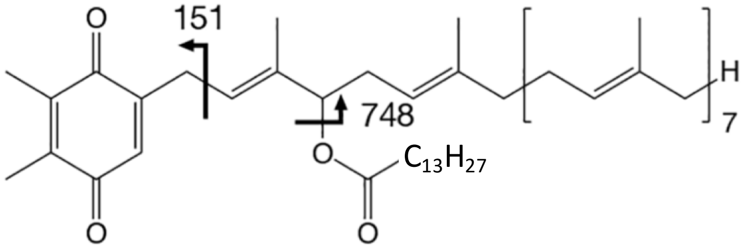

16:0-PQB

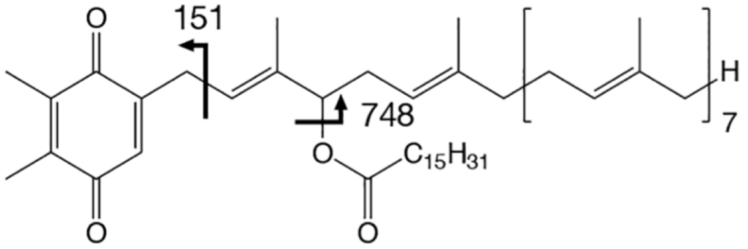

18:0-PQB

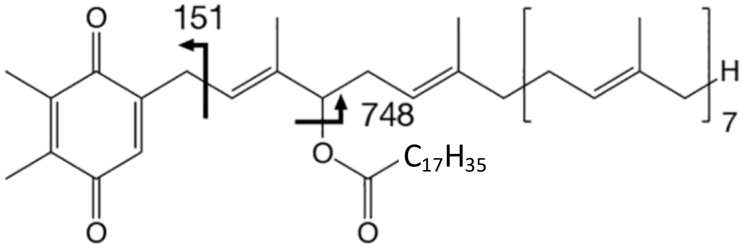

18:2-PQB

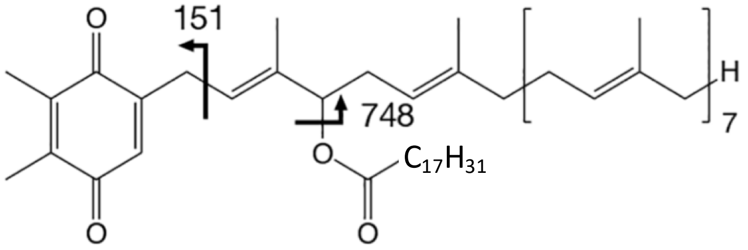

Figure S8 Fragmentation patterns of PQB species.
